# Emerging Evidence on Neutrophil Motility Supporting Its Usefulness to Define Vitamin C Intake Requirements

**DOI:** 10.3390/nu9050503

**Published:** 2017-05-16

**Authors:** Volker Elste, Barbara Troesch, Manfred Eggersdorfer, Peter Weber

**Affiliations:** DSM Nutritional Products AG, Human Nutrition and Health, P.O. 3255, CH-4002 Basel, Switzerland; barbara.troesch@dsm.com (B.T.); manfred.eggersdorfer@dsm.com (M.E.); peter.weber@dsm.com (P.W.)

**Keywords:** vitamin C, ascorbic acid, dietary reference value, immune function, neutrophil motility

## Abstract

Establishing intake recommendations for vitamin C remains a challenge, as no suitable functional parameter has yet been agreed upon. In this report, we review the emerging evidence on neutrophil motility as a possible marker of vitamin C requirements and put the results in perspective with other approaches. A recent in vitro study showed that adequate levels of vitamin C were needed for this function to work optimally when measured as chemotaxis and chemokinesis. In a human study, neutrophil motility was optimal at intakes ≥250 mg/day. Interestingly, a Cochrane review showed a significant reduction in the duration of episodes of common cold with regular vitamin C intakes in a similar range. Additionally, it was shown that at a plasma level of 75 µmol/L, which is reached with vitamin C intakes ≥200 mg/day, incidences of cardiovascular disease were lowest. This evidence would suggest that daily intakes of 200 mg vitamin C might be advisable for the general adult population, which can be achieved by means of a diverse diet. However, additional studies are warranted to investigate the usefulness of neutrophil motility as a marker of vitamin C requirements.

## 1. Introduction

Vitamin C is an essential micronutrient. As humans cannot produce it, the daily amount needed to ensure an adequate intake is defined in dietary reference values established in many countries around the globe. The guiding principle for the definition of dietary reference values for vitamin C, as for other essential micronutrients, has changed in the past few decades from only preventing deficiency syndromes to maintaining or even improving human health and ultimately reducing the risk of non-communicable diseases [1]. Scurvy—the clinical manifestation of vitamin C deficiency—develops when intake is below 10 mg/day for a prolonged period [2]. While it takes very little vitamin C to prevent an overt deficiency, the challenge is to define the daily intake required to maintain adequate health given the many metabolic processes that vitamin C is involved in.

The role of vitamin C in the human immune defense in particular is a widely researched field. However, the heterogeneity of study designs and the variability or even inconsistency of outcomes make it difficult to use these data as the basis for daily reference values. In the past, they were consequently deemed insufficient to reliably estimate the vitamin C requirement for apparently healthy individuals. The fact that vitamin C is actively accumulated in the leukocytes resulting in an up to 20 times higher concentration in neutrophils than in the plasma underscores its important role in immune defense. Agencies such as the Institute of Medicine (IOM) in North America used the near-maximal neutrophil concentration with minimal urinary loss to derive vitamin C reference values [1]. In that approach, the vitamin C intake required to near saturate the vitamin C concentration in neutrophils was employed to define the daily reference values.

Recently, additional evidence has emerged on the functional capacity of neutrophils relative to vitamin C concentration in vitro [3] and intakes in humans [4]. It is the aim of this contribution to review the potential of neutrophil motility as a possible marker for defining intake requirements for vitamin C in light of these recent findings. We will also discuss the current levels of vitamin C intakes and how to achieve appropriate intakes.

## 2. Physiologic Functions of Vitamin C in Human Health

**Figure 1:**
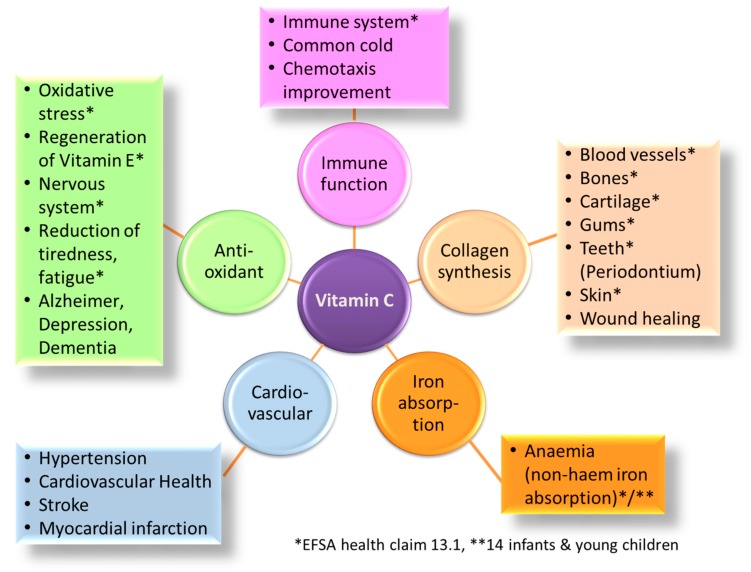
Summary of the functions of vitamin C and established health claims by European Food Safety Authority (EFSA), Article 13.1 and 14.

Vitamin C can be in the form of L-ascorbic acid and the oxidized form L-dehydroascorbic acid, and both are essential for a range of vital functions (Figure 1). Humans, as well as some other species such as monkeys, guinea pigs, some fish species, and birds, have lost L-gulonolactone oxidase, the enzyme catalyzing the last step in the synthesis of vitamin C, which makes them dependent on ample amounts of vitamin C from the diet [5,6].

Thanks to its reducing power, vitamin C mainly functions either as an antioxidant [7] or as a cofactor in enzymatic reactions [5]. As an antioxidant, it scavenges free radicals such as reactive oxygen species and reactive nitrogen species, turning them into less reactive molecules [8]. Through this mechanism, vitamin C protects proteins, lipids, and nucleic acids, and thus the body in general, from oxidative damage. Due to its antioxidant function, it contributes, for instance, to the protection of skin from UV irradiation [9,10] and is able to recycle other antioxidants such as vitamin E [11,12,13]. By doing so, it helps prevent low density lipoprotein (LDL) oxidation and protects cell lipids from peroxidation [12], thus it is essential for the proper function of the endothelium. In addition, vitamin C increases the bioavailability of non-hem iron by reducing it to the ferrous form, the only form which can be absorbed in the intestine [14,15]. It also increases iron solubility in the stomach and duodenum and reduces the likelihood of iron being affected by inhibitors of iron absorption [16].

As an essential cofactor of iron- and copper-dependent enzymes, vitamin C is involved as an electron donor in a range of catalytic redox-reactions [5]: It helps catalyze the synthesis of L-carnitine from L-lysine, which plays an important role in energy production via ß-oxidation in mitochondria [17,18,19]. However, in this reaction, its essentiality is discussed controversially, as it may be replaced by glutathione [20]. The synthesis of the catecholamine noradrenaline, a hormone and neurotransmitter, from dopamine by the enzyme dopamine β-monooxygenase also requires vitamin C [21]. Furthermore, the alpha-amidating monooxygenase needs it to increase the stability and activity of peptide hormones such as oxytocin and vasopressin [22]. Vitamin C also plays a role in the synthesis of hypoxia-inducible factor-1 alpha [23], and it is involved in tyrosine metabolism [24].

The vitamin C-dependent enzymes proline-hydroxylases and the lysine hydroxylase are essential for the synthesis of the proteoglycan collagen, which is the main molecule in connective tissues found for example in bone, periodontium, cartilage, skin, ligaments, tendons, and blood vessels [25,26]. Impaired collagen formation due to low vitamin C intake for several weeks leads to the typical symptoms of scurvy such as bleeding gums with the loss of teeth, malformation of bones, and weak blood vessels. This ultimately results in vasomotor instability and open wounds. For the formation and remodeling of bones, not only minerals are needed but also the organic matrix which contains up to 90% collagen produced in osteoblasts. Vitamin C deficiency will lead to bone loss or reduction in bone formation [27,28,29]. In addition, for wound healing, adequate amounts of vitamin C are needed because it is essential for fibroblast maturation, for the formation of cross-links between collagen fibers, and for angiogenesis [30,31], and thus it also has positive effects on pressure ulcers and burns [32].

Furthermore, vitamin C is a cofactor for the rate-limiting enzyme in bile acid synthesis, which may also enhance the expression of LDL receptors on hepatocytes, thus reducing LDL blood levels [33,34]. A recent review showed significant reductions in blood lipids after vitamin C supplementation in sub-populations with dyslipidemia or low vitamin C status at baseline [35]. This is in line with its positive impact towards a healthy cardiovascular system, reducing the prevalence of coronary heart disease [34] and stroke [36]. Its antioxidant effects reduce oxidative stress and enhance endothelial function through its effects on nitric oxide preservation and generation. Nitric oxide is a signaling molecule that activates endothelial and smooth muscle cells, which increases vasodilation, thus reducing blood pressure and preventing cardiovascular disease (CVD) [37]. Recent meta-analyses support this [38,39], showing that vitamin C improves endothelial function. Antihypertensive effects of vitamin C, particularly by reducing systolic blood pressure, have been shown in short-term trials [40]. Based on this evidence, it was concluded that vitamin C may be a useful nutritional intervention for the secondary prevention of CVD [2].

Furthermore, vitamin C plays an essential role in immune function, which is impaired by insufficient supply and re-established through supplementation [41,42]. It exerts its effect via the promotion of T-cell maturation by modulating the epigenetic regulation of gene expression as a cofactor of dioxygenases [43]. For the circulating immune cells, the importance of vitamin C is highlighted by the preferential uptake via active transport by the sodium-dependent vitamin C transporter located in their cell membranes, resulting in vitamin C concentrations from 20 to 60 times higher than in the surrounding plasma [44,45]. It is assumed that the high vitamin C concentration protects neutrophils from the reactive oxygen species (ROS) they generate to kill pathogens such as bacteria and viruses. Subsequently, extracellularly accumulated L-dehydroascorbic acid is rapidly transported back by glucose transporters and recycled to L-ascorbic acid [5]. Furthermore, it could be shown that vitamin C improves the immune function by influencing chemotaxis (CT) and chemokinesis (CK) of neutrophil leukocytes [46]. The enhancement in leukocyte motility by ascorbic acid goes along with its ability to assemble microtubule organelles [47]. These findings are supported by the observation that the vitamin C level is affected in people with infections, chronic diseases [48], and higher oxidative stress, as they lead to higher metabolic losses: e.g., during common cold [49] and in smokers, a 40% higher turnover is seen [50]. The multiple functions of vitamin C are also reflected by EFSA health claims (Figure 1).

## 3. Approaches Used to Define Vitamin C Requirements

Currently, the recommended intakes for adults tend to vary for the genders, but they also depend on the agency issuing them (Table 1). Moreover, most have additional allowances for periods of elevated needs, typically pregnancy and lactation [1,51,52,53,54,55,56,57,58]. In some recommendations, smokers are also advised to increase their intakes because their vitamin C status is lower than in non-smokers most likely due to the oxidative potential of the inhaled smoke [1,56,59]. Even though some differences exist in the data used to evaluate dietary requirements and the rationale applied in interpreting them, dietary reference values no longer simply aim at preventing overt deficiencies. They are meant to define intakes associated with optimal health for the majority of individuals—typically 95% to 97.5% of a specific age and gender group.

The first physiological marker used to define intake recommendations was the symptoms of scurvy. These could be prevented with daily intakes of around 10 mg, which led, with the inclusion of a safety margin of 30 to 50 mg, to the first set of recommendations for vitamin C of 60 mg per day [60,61]. However, even 20 years ago, this approach was challenged with the argument that a lack of overt deficiency did not necessarily indicate the adequacy of intake [61]. It was therefore suggested that recommended intakes should be at a level that assures optimal functioning of all processes requiring vitamin C, but still sufficiently below those known to provoke adverse effects [60,61]. As a consequence, bodies such as IoM and EFSA defined new levels of adequate intakes [1,56,62]. Moreover, they discussed potential biological markers of physiological functions, such as health outcomes associated with vitamin C intake. However, it was concluded that these were insufficiently established, and consequently the recommendations were still based on indicators of vitamin C status.

EFSA determined the Average Requirement (AR) for healthy adults based on the vitamin C intake that balanced losses as metabolic and urinary losses and the quantity of vitamin C required for the replacement of these losses to metabolic losses and maintained fasting plasma ascorbate concentrations at about 50 μmol/L. This led them to propose daily vitamin C intakes such as the Population Reference Intake (PRI) of 110 mg and 95 mg for healthy adult men and women, respectively [62]. The German-speaking countries adapted their joint reference values to the EFSA recommendations [57]. IoM chose a slightly different approach by using the near-maximal neutrophil concentration with minimal urinary excretion of ascorbate to provide antioxidant protection, which led them to define a Recommended Daily Allowances (RDA) of 90 mg for adult men and 75 mg for adult women [1]. However, both IoM and EFSA highlighted the need to establish an accurate, specific, and easily measurable functional marker for vitamin C (e.g., IoM [1]).

Given the range of functions vitamin C has in the human body, there are many putative functional markers. However, their lack of specificity was one of the main reasons why they were deemed unsuitable as a basis to define vitamin C intake recommendations [63]: Assessing hepatic enzyme systems by measuring cholesterol concentration and detoxification has been proposed [64], but it is influenced by a range of factors and is therefore not specific enough to define vitamin C status. The same applies to measuring DNA oxidation as an indicator of DNA damage [63]. Collagen turnover measured as hydroxyproline excretion was another potential candidate [65]. Unfortunately, the high inter- and intra-individual variation in the response to varying vitamin C intakes reduces its usefulness as a biomarker to define intake recommendations [66]. Given the inverse relationship between vitamin C and blood pressure, this has been discussed as a putative physiological biomarker, but again, it is not sufficiently specific for vitamin C status [67,68,69]. Furthermore, the urinary excretion of vitamin C has been proposed as a sign of adequate intake. However, this is not a functional marker and reflects only one aspect of vitamin C plasma homeostasis. It could be shown that the bioavailability is complete for 200 mg vitamin C as a single dose [45]. These examples show that a putative marker needs to be reasonably specific to vitamin C status, sensitive to changes in intake within the relevant range, and reliably measurable.

## 4. New Insights Support Reassessment of Current Vitamin C RDAs

Recently published data might be able to shed some light on the question of suitable indicators and consequently enable us to define more appropriate recommendations for daily intakes. The effect of vitamin C on motility in neutrophils seems to be a promising candidate for such a marker [70]. In the following paragraphs, we review the suitability for the use of this marker and how it fits with the well-established knowledge.

Neutrophils are the most abundant type of leukocytes (40% to 75%) and play an important role in the innate immune system [71]. The cells are highly motile and are able to migrate from the blood into the affected tissues in a process initiated and orchestrated by chemoattractants such as pathogen-derived products or host-derived factors [72]. Chemotaxis (CT) and chemokinesis (CK) describe this movement: while the former is directional, the later consists of random movement [70]. Neutrophils contain high concentrations of vitamin C compared to plasma levels [45], and it is thought that they function best if adequate amounts of vitamin C are available [47]. It has, for example, been shown that inadequate intakes could impair CT in guinea pig leukocytes [73]. The underlying mechanism is thought to be the ability of vitamin C to promote the assembly of microtubule organelles [47].

Similar effects could be shown in a recent in vitro study investigating the impact of vitamin C on CT and CK in cell cultures [3]. It showed that extracellular vitamin C significantly increased CT in vitamin C-preloaded peripheral blood leukocytes, which predominantly consist of neutrophils [3]. Furthermore, vitamin C at physiological concentrations also affected CK, indicating that vitamin C enhances directional and random migration at concentrations comparable to those observed in plasma [3]. This could be seen as a further indication that neutrophil function could be a suitable functional marker to define vitamin C intake. Nevertheless, previous clinical studies investigating the role of vitamin C on neutrophil chemotaxis showed inconsistent effects, and they did not allow for the estimation of the vitamin C requirement for apparently healthy individuals reliably [1]. Therefore, further well designed human studies are warranted.

The findings of the in vitro studies are corroborated by a human study investigating the effect of vitamin C supplementation on the function of neutrophils [4]: Healthy young men with suboptimal plasma vitamin C status (<50 µmol/L) were supplemented with vitamin C rich kiwi fruits (~260 mg/day vitamin C) for four weeks. This is in line with the postulation that such studies should use the baseline vitamin status below a defined threshold as inclusion criteria [74]. Despite the relatively small sample size of 12 participants, the plasma and neutrophil vitamin C content as well as chemotaxis of neutrophils and superoxide generation increased significantly [4].

These results suggest that supplementation of vitamin C from kiwi fruits is associated with the improvement of important neutrophil functions and consequently enhanced immunity. Given the study design, it cannot be excluded that other components of the kiwi fruits contributed to this effect. This needs to be confirmed with an intervention using vitamin C supplements at different doses and a placebo group including more subjects to prove which is the right dose. However, based on the results from the in vitro study, it is very likely that vitamin C was the active compound. While neutrophil saturation was already reached at intakes of 100 mg/day [45], it seems that for optimal maturation and functioning of these cells, intakes of ≥200 mg/day result in additional benefits [3,4]. This is supported by the results from a study showing that intakes of ~110 mg (~60 mg from the diet plus ~53 mg vitamin C from one-half kiwifruit) resulted in saturated neutrophil levels, but not saturated plasma levels. [75]. When they increased the dose of kiwi fruit to two per day with a total vitamin C intake of around 210 mg, plasma vitamin C levels, as well as urinary excretion, further increased [76]. This is thought to be sufficient to achieve a plasma level of ≥70 µmol/L, which should be reached to ensure optimal immune function by the neutrophil leucocytes. Moreover, this is also within the range where the human sodium-dependent vitamin C transporter 2, which is responsible for the uptake of vitamin C into target tissues, is at maximum velocity [77]. In addition, this level of intake enables optimal vitamin C supply in all stages of the neutrophil development and therefore ensures maximal functions of these short-lived immune cells. Importantly, intakes ≥200 mg/day were not associated with any adverse outcomes [45] and are well within the range of <2000 mg/day, which are considered safe by IoM [1]. EFSA considers that supplemental daily doses of vitamin C up to about 1 g are not associated with adverse gastrointestinal effects, and an increased risk of kidney stones was not found in individuals with habitual intakes of 1.5 g/day [78].

For healthy young women, the intakes required for plasma and plasma saturation were slightly lower (100 to 200 mg/day) [79]. However, the authors of this subsequent pharmacokinetics study still concluded that vitamin C intakes of 200 mg from foods are probably required, as bioavailability might be lower from whole fruits and vegetables compared to supplements [79]. Further studies in women are needed to assess the optimal dose of the vitamin for neutrophil function. Moreover, the requirements might also increase with age and possibly body weight as well, given the increased level of inflammation and consequently oxidative stress accompanying both [80,81]. To adapt the recommendation to different groups requires further evaluations.

## 5. The Improvement of Neutrophil Function by Vitamin C in a Broader Human Health Perspective

### 5.1. Common Cold

Given its importance for the immune system, improved vitamin C status can be expected to translate into clinical endpoints when faced with infections. This was assessed in a recent Cochrane review investigating the effect of vitamin C intake on the common cold in adults and children [82]. In this meta-analysis, no significant effect of supplementation with between 200 mg and 2000 mg of vitamin C daily on the incidence of common cold was found. However, as is often the case in nutritional randomized controlled trials, the placebo group did not have zero intake of vitamin C, as the participants’ diets provide potentially significant amounts of the nutrient in question [83]. Therefore, it is crucial to enroll subjects with hypovitaminosis (plasma vitamin C <50 µmol/L) for such trials [74] to be able to work with an approximation of an actual placebo group. Moreover, in a few studies, the ‘placebo’ groups also received 50 to 70 mg/day vitamin C for ethical reasons [82]. The fact that in a subgroup analysis of persons with ‘acute physical activity’ and consequently, higher requirements, vitamin C supplementation reduced the number of incidents by half (Risk Ratio 0.48, 95% Confidence Interval 0.35 to 0.64) supports this interpretation. Interestingly, this was not the case in those with long-term physical stress.

Despite these limitations, it was found that vitamin C supplementation of ≥200 mg significantly reduced the duration of common cold symptoms: In children, the effect was reduced by ~14% and in adults, it was reduced by nearly 8% [82]. Interestingly, it was found that in children, it increased to 18% if only studies supplementing ≥1000 mg were included. This might indicate that during an acute infection, higher intakes could be beneficial—even though this effect was not seen in adults. Neutrophils are under increased oxidative stress during an infection, and it has been shown that vitamin C concentrations greatly increase when they are activated (see the review by Padayatty and Levine [5]). Moreover, supplementation led to a modest but significant reduction in the days that the participants missed from work or school due to the common cold.

**Figure 2:**
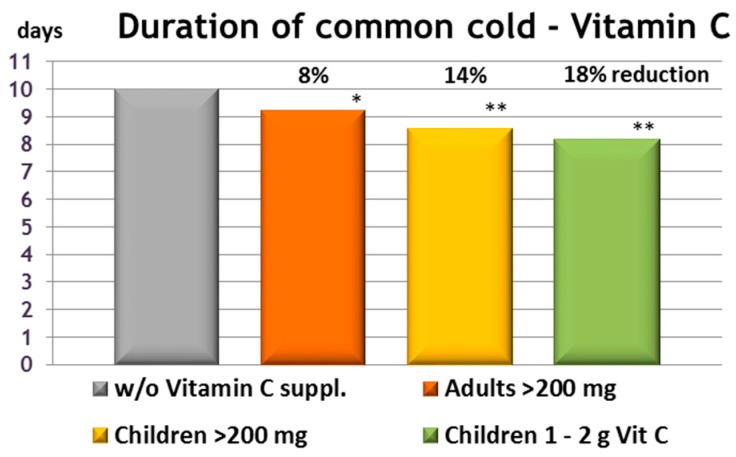
Duration of the common cold. Effect of regular, prophylactic supplementation of vitamin C (≥200 mg/day) on the duration of the common cold, assuming a 10-day illness in adults * (17 trials, 8%; *p* = 0.0002) and in children ** (total 14 trials, 14% for ≥200 mg/day and 10 trials, 18% for 1 to 2 g/day; *p* < 0.0001), adapted from Hemila and Chalker, 2013 [82].

On average, episodes of common colds last around 10 days [84] and children tend to have 3 to 5 per year, while for adults it is 1 to 2. Consequently, it can be estimated that an adequate supply with vitamin C can reduce the days spent being ill by 4 to 6 and 1 to 2 for children and adults, respectively (see Figure 2). In addition, the meta-analysis reported a reduction in the severity of the common cold thanks to supplementation with vitamin C, even though the interpretation of this is difficult due to the wide range of the definitions of ‘severity‘ used in the various studies [82].

The findings of studies starting vitamin C supplementation only after the onset of symptoms of common cold were equivocal [82]. This is not surprising, considering that neutrophils are crucial in recognizing an infection and initiating an immune response to fight it. They should therefore already be functioning well before a cold is caught. Given their short half-life, only regular intake at adequate levels can ensure that sufficient mature neutrophils are produced. Marginal vitamin C levels, on the other hand, reduce the CT and CK of the neutrophils, which leads to a slowed immune response.

### 5.2. Non-Communicable Diseases

Vitamin C is thought to play an important role in the prevention of non-communicable diseases such as CVD and cancer [48,85]. One difficulty is that randomized controlled trials are not necessarily suitable for detecting such a relationship between a nutrient and a disease [83]. One review found that none of the available studies used low plasma vitamin C concentrations as inclusion criteria, and that the participants of a large majority of these trials (34 out of 35) were unlikely to show a benefit given their baseline plasma concentrations [74].

The link between inadequate vitamin C intake and non-communicable disease is best documented for CVD (for a detailed review of the evidence, see Frei et al., 2012 [85] and Moser et al., 2016 [86]). Given that atherosclerosis is an inflammatory disease [87], it seems likely that vitamin C plays an important role in protecting against it: Vitamin C depletion is thought to increase the susceptibility of LDL cholesterol to oxidation, a risk factor for CVD [88]. However, it is now equally recognized that reactive oxygen species formed by the inflammatory response in an existing atherosclerotic lesion may in turn reduce vitamin C antioxidant levels [87]. A recent review of epidemiologic studies supports the finding that endothelial function and lipid profiles, especially in subjects with low plasma levels, are improved by vitamin C. This is in line with large prospective studies that have shown an inverse relationship between plasma vitamin C status and the risk of CVD [88,89,90,91,92]. Also, Langlois and colleagues [93] showed a relationship between vitamin C concentration and the severity of atherosclerosis and inflammation in peripheral artery disease patients. Moreover, there is evidence from a meta-analysis of randomized controlled trials that vitamin C supplementation has a beneficial effect on blood pressure [40]. Furthermore, sub-group analysis of a recent meta-analysis revealed that vitamin C supplementation reduced LDL cholesterol in healthy participants and, triglycerides are reduced and HDL cholesterol is significantly increased in diabetics. Furthermore, greater effects of vitamin C supplementation in lowering total cholesterol and triglycerides could be shown in those with higher concentrations of these lipids at baseline and the HDL cholesterol increase was greater in participants with lower baseline plasma concentrations of vitamin C, while the overall effects were not significant [39].

Frei [85] argues that the scientific evidence from metabolic, pharmacokinetic, epidemiologic, and intervention studies strongly advocates for an increase of the recommended daily intake to ≥200 mg/day to minimize the risk of negative health effects. Moreover, if the vitamin C content of a healthy, balanced diet in line with guidelines to prevent non-communicable diseases is estimated, it adds up to values slightly above 200 mg/day [94]. Even though these findings relate to different functions of vitamin C, they indicate optimal intakes in a range similar to that suggested by neutrophil motility, thereby strengthening the proposed recommendations. In addition, even though fraught with the same problems as for the other health outcomes, there is some evidence that maintaining healthy vitamin C levels might offer some protection against age-related cognitive decline and Alzheimer’s disease [95]. This is not surprising, in the light of the mounting evidence for the role of CVD [96] and oxidative stress in the development of Alzheimer’s disease [97]. Given the number of people affected by hypertension, CVD, dementia, and cancer, defining recommendations with the highest risk reduction for these diseases is of paramount importance.

## 6. Vitamin C Status in the General Population

Proposing to increase the dietary intake recommendations for vitamin C raises the question of whether and how these can be achieved by the general population. Based on the typical food-based dietary recommendations, even the increased intakes of ≥200 mg/day should in theory not cause a problem: Many countries translated the WHO recommendation of ≥400 g of fruits and vegetables, excluding potatoes, cassava, and other tubers, per day [98] into at least five daily servings of such foods. As many of these fruits and vegetables provide significant amounts of vitamin C per average serving (see Table 2), it is feasible to supply ≥200 mg/day of the vitamin via a balanced diet. This is particularly the case if at least one item with high vitamin C levels (e.g., orange juice) is included in the daily diet and preparation techniques such as steam cooking are used that reduce the loss of vitamin C [99]. In addition, other foods also contribute important amounts: A relatively recent German dietary survey reported that the main dietary sources for vitamin C were fruits and fruit products, non-alcoholic beverages, and vegetables [100]. However, potatoes, meat, and meat products such as sausages, as well as dairy products contributed to an important, but lesser, degree [100].

A study in Greece showed that adults who did not meet the recommended daily intakes for fruits or vegetables had a higher risk of inadequate vitamin C intakes [101]. Not surprisingly, those who complied with this specific dietary recommendation tended to have adequate amounts in their diet [101]. In line with this, a study in Switzerland compared vitamin C intakes in omnivores, vegetarians, and vegans, and found mean intakes of 94 mg/day, 158 mg/day, and 239 mg/day, respectively [102]. The corresponding plasma C levels were ~55 µmol/L, ~69 µmol/L, and ~72 µmol/L, respectively. This ties in nicely with the estimate that intakes of ≥200 mg/day achieve plasma levels in the desirable range of >70 µmol/L [45]. Even though the data on foods consumed was not reported in the Swiss study, it can be assumed that the increased intakes of fruits and vegetables in vegetarians and vegans reported elsewhere [103] is reflected in this data.

However, people tend not to follow food-based dietary advice and tend to eat too much of what they should reduce and not enough of the foods that they are encouraged to eat [104]. This is no different in the case of fruits and vegetables, as seen in a recent study: 58% to 88% of adults around the world did not consume the recommended five servings per day [105]. A recent survey from Switzerland showed that only 13% consume the recommended five servings per day [106]. This is in line with an earlier study, which reported that less than 25% of the general adult population in low- and middle-income countries actually followed the recommendation of five portions of fruits and vegetables per day [107]. On the bright side, data from France shows an increase in the consumption of fresh fruits and vegetables, accompanied by a parallel increase in vitamin C intakes, albeit from comparatively low levels (mean intakes for adults <100 mg/day) [108].

This puts the French into the middle range of intakes within Europe: The European Nutrition and Health Survey reports mean vitamin C intakes ranging from ~60 mg to ~153 mg [110]. However, the informative value of mean intakes is limited when assessing the adequacy of intake of a population: despite the comparatively high mean intake reported for Germany (153 mg/day for adults) [110], half the adult population has vitamin C intake below 100 mg/day, which was the recommendation at the time of the survey [59,111,112]. Using a lower level of 60 mg/day and 50 mg/day for men and women, respectively, the European survey reports on 8% to 40% of adults with inadequate intakes [113], and similar rates were reported in the U.S. [114]. Unfortunately, for many countries, only the information on mean intakes is available. However, as the mean intakes are in a similar range as those reported in the surveys referred to above, it can be assumed that a similar problem exists in many—also affluent—parts of the world: In Japan, median intakes of 60 mg and 100 to 115 mg were reported for the age group of 15 to 49 and ≥50 years, respectively [115]. Similarly, mean intakes in South Korea were 116 mg in men and 105 mg in women [116].

Dietary supplements also play an important role in the provision of vitamin C: supplement users across all age groups were found to have higher serum concentrations and lower risk of deficiency than non-users [117]. In the U.S., the proportion of the general population (aged ≥2 years) with intakes below the *Estimated Average Requirement* for vitamin C decreased from 46% to 25% if fortified foods and supplements were taken into account [118]. However, supplement use in Europe is less common, and there is a strong north-to-south gradient, with >40% and 5%, respectively, consuming some type of dietary supplement [113]. Still, in Germany, vitamin C supplements are those used most frequently, and around 10% reported taking them [111]. Similarly, it was among the three most commonly used supplements in a study across Europe [119], and supplements can therefore be assumed to play an important role as dietary sources for the vitamin.

The contribution of different foods to vitamin C intake depends on a range of factors such as variety, maturity of the fruit or vegetable when harvested, and the climate where it grew [120,121,122], but also on the processing technique involved [63,109]. This makes it difficult to extrapolate the actual status from dietary intake data. However, serum vitamin C concentrations—a more direct marker of vitamin C status—show a similar picture: An analysis in Canada classified 14% of adults as vitamin C deficient and a further 33% as having sub-optimal serum levels [123]. In the U.S., similar rates for vitamin C deficiency were measured when serum levels were reported in the 1988 to 1994 survey [124], but the prevalence was found to decrease to around 7% in 2003 to 2004 [2]. However, given that persons on low incomes were at increased risk of deficiency [2], it is very likely that the economic crisis, and the consequent increase in poverty and food insecurity [125], has reversed this trend. Moreover, there was a trend towards lower levels for obese persons, which reached significance for women, but not for men [2]. Given the dramatic increase in the prevalence of obesity reported [126], this is worrying, even though it is not clear whether there is a causal link.

In summary, it can be said that the available evidence indicates that even in affluent societies, a significant proportion of the population does not achieve adequate vitamin C status, even as defined by the current recommendations. Increasing the recommended intake to levels more in line with our current understanding of optimal status will further increase the gap between actual intakes and what is regarded as being compatible with optimal health. This might increase the motivation to optimize vitamin C intake either by food fortification or the use of supplements.

## 7. Conclusions

In light of the many functions that vitamin C has in the body, a range of putative biomarkers were proposed, but they have been rejected due to shortcomings such as lack of specificity (See above). Up to now, no functional biomarker was identified that could be used as a basis to define the dietary intake recommendations for vitamin C. Even though scientific bodies such as IoM argued that such an indicator is needed when they revised their recommendations, they concluded that none have been identified yet [1]. Based on the findings of an in vitro [3] and a human intervention study [4], we propose to investigate further neutrophil motility as such a functional marker.

Combined with the established knowledge from pharmacokinetic, observational, and intervention studies, they indicate that current recommended intakes are set too low and that an increase to ≥200 mg/day would be beneficial for the functioning of the immune system. The importance of vitamin C for the immune system was also recognized by the EFSA Panel on Dietetic Products, Nutrition, and Allergies by granting the health claim that vitamin C contributes to a normal function of the immune system [127]. Moreover, such intakes are sufficient to keep plasma vitamin C levels at >70 µmol/L—the range which is associated with plasma saturation [45], but also with reduced risk of CVD [74].

Further well-designed studies in humans are needed to validate neutrophil motility as a functional marker of vitamin C sufficiency and immune function. Moreover, existing questions on the essentiality of adequate vitamin C intakes in the prevention of a range of non-communicable diseases such as CVD, but also cancer and dementia, need to be resolved. This requires large prospective cohort studies, but also randomized controlled trials in participants with low baseline plasma vitamin C levels. In addition to the general population, studies should also address sub-populations, which might have elevated needs due to their genotype or other characteristics, such as obesity, smoking, or increased physical activity.

Even though ≥200 mg/day vitamin C could be achieved via a balanced diet in line with the guidelines for the prevention of non-communicable diseases, significant proportions of the population do not achieve even the current recommendations. Consequently, methods need to be found to increase vitamin C intake in the general population—ideally via increased intakes of fruits and vegetables, given the benefits of such foods beyond their vitamin C content. However, as changing people’s food habits is notoriously difficult, fortified foods or supplements might provide a more realistic solution at least in the short term.

## Figures and Tables

**Table 1:** Examples for a wide range of recommended daily intakes for vitamin C in adults (≥19 years) in different countries and regions.

Country	Men (mg)	Women (mg)
Germany, Austria, and Switzerland [56,59]	110	95
United States [1]	90	75
United Kingdom [52]	40	40
Australia and New Zealand [54]	45	45
Japan [58]	100	100
Philippines [55]	75	70
Singapore [57]	105	85
South Africa [51]	90	90
FAO/WHO [53]	45	45

FAO: Food and Agriculture Organization of the United Nations; WHO: World Health Organization

**Table 2:** Composition of a range of raw fruits and vegetables (data from the U.S. Department of Agriculture [109]).

Food	Content per 100 g (mg)	Unit	Content per Unit (mg)
Vegetables			
Red pepper	128	1 piece (119 g)	152
Green pepper	80	1 piece (119 g)	96
Broccoli	89	1 cup ^1^ (91 g)	81
Brussels sprouts	85	1 cup ^1^ (88 g)	75
Cabbage	37	1 cup ^1^ (89 g)	33
Cauliflower	48	1 cup ^1^ (107 g)	52
Tomato	14	1 piece (123 g)	17
Green peas	40	1 cup ^1^ (145 g)	58
Fruits			
Orange	53	1 piece (96 g)	70
Kiwi	93	1 piece (69 g)	64
Mango	36	1 piece ^2^ (336 g)	122
Strawberry	59	1 cup ^1^ (144 g)	85
Cantaloupe melon	37	1 wedge (69 g)	25
Grapefruit	33	1 piece (118 g)	39

^1^ 1 cup ≈ 2.4 dL; ^2^ without refuse.

## References

[1] Institute of Medicine (2000). Dietary Reference Intakes of Vitamin C, Vitamin E, Selenium, and Carotenoids.

[2] Schleicher R.L., Carroll M.D., Ford E.S., Lacher D.A. (2009). Serum vitamin C and the prevalence of vitamin C deficiency in the United States: 2003–2004 National health and nutrition examination survey (NHANES). Am. J. Clin. Nutr..

[3] Schwager J., Bompard A., Weber P., Raederstorff D. (2015). Ascorbic acid modulates cell migration in differentiated HL-60 cells and peripheral blood leukocytes. Mol. Nutr. Food Res..

[4] Bozonet S.M., Carr A.C., Pullar J.M., Vissers M.C. (2015). Enhanced human neutrophil vitamin C status, chemotaxis and oxidant generation following dietary supplementation with vitamin C-rich sungold kiwifruit. Nutrients.

[5] Padayatty S.J., Levine M. (2016). Vitamin c: The known, the unknown, and goldilocks. Oral Dis..

[6] Linster C.L., Van Schaftingen E. (2007). Vitamin c. Biosynthesis, recycling and degradation in mammals. FEBS J..

[7] Buettner G.R., Jurkiewicz B.A. (1996). Catalytic metals, ascorbate and free radicals: Combinations to avoid. Radiat. Res..

[8] Valko M., Leibfritz D., Moncol J., Cronin M.T., Mazur M., Telser J. (2007). Free radicals and antioxidants in normal physiological functions and human disease. Int. J. Biochem. Cell Biol..

[9] Swindells K., Rhodes L.E. (2004). Influence of oral antioxidants on ultraviolet radiation-induced skin damage in humans. Photodermatol. Photoimmunol. Photomed..

[10] Eberlein-Konig B., Placzek M., Przybilla B. (1998). Protective effect against sunburn of combined systemic ascorbic acid (vitamin C) and d-alpha-tocopherol (vitamin E). J. Am. Acad. Dermatol..

[11] Buettner G.R. (1993). The pecking order of free radicals and antioxidants: Lipid peroxidation, alpha-tocopherol, and ascorbate. Arch. Biochem. Biophys..

[12] Sharma M.K., Buettner G.R. (1993). Interaction of vitamin C and vitamin E during free radical stress in plasma: An esr study. Free Radic. Biol. Med..

[13] Benzie I., Strain J.J. (1999). Effect of vitamin C supplementation on concentrations of vitamins C and E in fasting plasma. Asia Pac. J. Clin. Nutr..

[14] Forth W., Rummel W. (1973). Iron absorption. Physiol. Rev..

[15] Lynch S.R., Cook J.D. (1980). Interaction of vitamin C and iron. Ann. N. Y. Acad. Sci..

[16] Scheers N., Andlid T., Alminger M., Sandberg A.S. (2010). Determination of Fe^2+^ and Fe^3+^ in aqueous solutions containing food chelators by differential pulse anodic stripping voltammetry. Electroanalysis.

[17] Fritz I.B. (1963). Carnitine and its role in fatty acid metabolism. Adv. Lipid Res..

[18] Fritz I.B., Yue K.T. (1963). Long-chain carnitine acyltransferase and the role of acylcarnitine derivatives in the catalytic increase of fatty acid oxidation induced by carnitine. J. Lipid Res..

[19] Ramsay R.R., Gandour R.D., van der Leij F.R. (2001). Molecular enzymology of carnitine transfer and transport. Biochim. Biophys. Acta.

[20] Furusawa H., Sato Y., Tanaka Y., Inai Y., Amano A., Iwama M., Kondo Y., Handa S., Murata A., Nishikimi M. (2008). Vitamin C is not essential for carnitine biosynthesis in vivo: Verification in vitamin C-depleted senescence marker protein-30/gluconolactonase knockout mice. Biol. Pharm. Bull..

[21] Rush R.A., Geffen L.B. (1980). Dopamine beta-hydroxylase in health and disease. Crit. Rev. Clin. Lab. Sci..

[22] Prigge S.T., Kolhekar A.S., Eipper B.A., Mains R.E., Amzel L.M. (1999). Substrate-mediated electron transfer in peptidylglycine alpha-hydroxylating monooxygenase. Nat. Struct. Biol..

[23] Dengler V.L., Galbraith M.D., Espinosa J.M. (2014). Transcriptional regulation by hypoxia inducible factors. Crit. Rev. Biochem. Mol. Biol..

[24] Lindblad B., Lindstedt G., Lindstedt S. (1970). The mechanism of enzymic formation of homogentisate from *p*-hydroxyphenylpyruvate. J. Am. Chem. Soc..

[25] Kukkola L., Hieta R., Kivirikko K.I., Myllyharju J. (2003). Identification and characterization of a third human, rat, and mouse collagen prolyl 4-hydroxylase isoenzyme. J. Biol. Chem..

[26] Prockop D.J., Kivirikko K.I. (1995). Collagens: Molecular biology, diseases, and potentials for therapy. Annu. Rev. Biochem..

[27] Aghajanian P., Hall S., Wongworawat M.D., Mohan S. (2015). The roles and mechanisms of actions of vitamin C in bone: New developments. J. Bone Miner. Res..

[28] Hasegawa T., Li M., Hara K., Sasaki M., Tabata C., de Freitas P.H., Hongo H., Suzuki R., Kobayashi M., Inoue K. (2011). Morphological assessment of bone mineralization in tibial metaphyses of ascorbic acid-deficient ods rats. Biomed. Res..

[29] Masse P.G., Jougleux J.L., Tranchant C.C., Dosy J., Caissie M., Coburn S.P. (2010). Enhancement of calcium/vitamin D supplement efficacy by administering concomitantly three key nutrients essential to bone collagen matrix for the treatment of osteopenia in middle-aged women: A one-year follow-up. J. Clin. Biochem. Nutr..

[30] Blass S.C., Goost H., Tolba R.H., Stoffel-Wagner B., Kabir K., Burger C., Stehle P., Ellinger S. (2012). Time to wound closure in trauma patients with disorders in wound healing is shortened by supplements containing antioxidant micronutrients and glutamine: A prct. Clin. Nutr..

[31] Thompson C., Fuhrman M.P. (2005). Nutrients and wound healing: Still searching for the magic bullet. Nutr. Clin. Pract..

[32] Stechmiller J.K. (2010). Understanding the role of nutrition and wound healing. Nutr. Clin. Pract..

[33] McRae M.P. (2008). Vitamin C supplementation lowers serum low-density lipoprotein cholesterol and triglycerides: A meta-analysis of 13 randomized controlled trials. J. Chiropr. Med..

[34] Hallfrisch J., Singh V.N., Muller D.C., Baldwin H., Bannon M.E., Andres R. (1994). High plasma vitamin C associated with high plasma HDL- and HDL2 cholesterol. Am. J. Clin. Nutr..

[35] Ashor A.W., Siervo M., van der Velde F., Willis N.D., Mathers J.C. (2016). Systematic review and meta-analysis of randomised controlled trials testing the effects of vitamin C supplementation on blood lipids. Clin. Nutr..

[36] Simon J.A., Hudes E.S., Browner W.S. (1998). Serum ascorbic acid and cardiovascular disease prevalence in US. Adults. Epidemiology..

[37] May J.M., Harrison F.E. (2013). Role of vitamin C in the function of the vascular endothelium. Antioxid. Redox Signal..

[38] Ashor A.W., Lara J., Mathers J.C., Siervo M. (2014). Effect of vitamin C on endothelial function in health and disease: A systematic review and meta-analysis of randomised controlled trials. Atherosclerosis.

[39] Ashor A.W., Siervo M., Lara J., Oggioni C., Afshar S., Mathers J.C. (2015). Effect of vitamin C and vitamin E supplementation on endothelial function: A systematic review and meta-analysis of randomised controlled trials. Br. J. Nutr..

[40] Juraschek S.P., Guallar E., Appel L.J., Miller E.R. (2012). Effects of vitamin C supplementation on blood pressure: A meta-analysis of randomized controlled trials. Am. J. Clin. Nutr..

[41] Wintergerst E.S., Maggini S., Hornig D.H. (2006). Immune-enhancing role of vitamin C and zinc and effect on clinical conditions. Ann. Nutr. Metab..

[42] Pike J., Chandra R.K. (1995). Effect of vitamin and trace element supplementation on immune indices in healthy elderly. Int. J. Vitam. Nutr. Res..

[43] Manning J., Mitchell B., Appadurai D.A., Shakya A., Pierce L.J., Wang H., Nganga V., Swanson P.C., May J.M., Tantin D. (2013). Vitamin C promotes maturation of t-cells. Antioxid. Redox Signal..

[44] Washko P., Rotrosen D., Levine M. (1989). Ascorbic acid transport and accumulation in human neutrophils. J. Biol. Chem..

[45] Levine M., Conry-Cantilena C., Wang Y., Welch R.W., Washko P.W., Dhariwal K.R., Park J.B., Lazarev A., Graumlich J.F., King J. (1996). Vitamin C pharmacokinetics in healthy volunteers: Evidence for a recommended dietary allowance. Proc. Natl. Acad. Sci. USA.

[46] Vohra K., Khan A.J., Telang V., Rosenfeld W., Evans H.E. (1990). Improvement of neutrophil migration by systemic vitamin c in neonates. J. Perinatol..

[47] Boxer L.A., Vanderbilt B., Bonsib S., Jersild R., Yang H.H., Baehner R.L. (1979). Enhancement of chemotactic response and microtubule assembly in human leukocytes by ascorbic acid. J. Cell. Physiol..

[48] Carr A.C., Frei B. (1999). Toward a new recommended dietary allowance for vitamin C based on antioxidant and health effects in humans. Am. J. Clin. Nutr..

[49] Hume R., Weyers E. (1973). Changes in leucocyte ascorbic acid during the common cold. Scott. Med. J..

[50] Lykkesfeldt J., Loft S., Nielsen J.B., Poulsen H.E. (1997). Ascorbic acid and dehydroascorbic acid as biomarkers of oxidative stress caused by smoking. Am. J. Clin. Nutr..

[51] The Nutrition Information Centre of the University of Stellenbosch. http://www.sun.ac.za/english/faculty/healthsciences/nicus/Pages/Vitamin-C.aspx.

[52] Department of Health (1991). Dietary Reference Values for Food, Energy and Nutrients for the United Kingdom in Report on Health and Social Subjects.

[53] Food and Agriculture Organization, World Health Organization (2002). Human Vitamin and Mineral Requirements.

[54] Australian National Health and Medical Research Council, New Zealand Ministry of Health Nutrient Reference Values for Australia and New Zealand. https://www.nrv.gov.au/nutrients/vitamin-c.

[55] Barba C.V., Cabrera M.I. (2008). Recommended energy and nutrient intakes for Filipinos 2002. Asia Pac. J. Clin. Nutr..

[56] German Nutrition Society (2015). New reference values for vitamin C intake. Ann. Nutr. Metab..

[57] Health Promotion Board Recommended Dietary Allowances. http://www.hpb.gov.sg/HOPPortal/health-article/2652.

[58] National Institute of Health and Nutrition Dietary Reference Intakes for Japanese (2015). http://www.mhlw.go.jp/file/06-Seisakujouhou-10900000-Kenkoukyoku/overview.pdf.

[59] Deutsche Gesellschaft für Ernährung, Österreichische Gesellschaft für Ernährung, Schweizerische Gesellschaft für Ernährung, Schweizerische Vereinigung für Ernährung (2008). Referenzwerte für die Nährstoffzufuhr.

[60] Levine M., Dhariwal K.R., Washko P.W., Welch R.W., Wang Y. (1993). Cellular functions of ascorbic acid: A means to determine vitamin c requirements. Asia Pac. J. Clin. Nutr..

[61] Levine M., Dhariwal K.R., Welch R.W., Wang Y., Park J.B. (1995). Determination of optimal vitamin C requirements in humans. Am. J. Clin. Nutr..

[62] EFSA NDA Panel (2013). Scientific opinion on dietary reference values for Vitamin C. EFSA J..

[63] Benzie I.F. (1999). Vitamin C: Prospective functional markers for defining optimal nutritional status. Proc. Nutr. Soc..

[64] Ginter E. (1989). Ascorbic acid in cholesterol metabolism and in detoxification of xenobiotic substances: Problem of optimum vitamin C intake. Nutrition.

[65] Bates C.J. (1977). Proline and hydroxyproline excretion and vitamin C status in elderly human subjects. Clin. Sci. Mol. Med..

[66] Hevia P., Omaye S.T., Jacob R.A. (1990). Urinary hydroxyproline excretion and vitamin C status in healthy young men. Am. J. Clin. Nutr..

[67] Rodrigo R., Prat H., Passalacqua W., Araya J., Bachler J.P. (2008). Decrease in oxidative stress through supplementation of vitamins C and E is associated with a reduction in blood pressure in patients with essential hypertension. Clin. Sci. (Lond.).

[68] Bendich A., Langseth L. (1995). The health effects of vitamin c supplementation: A review. J. Am. Coll. Nutr..

[69] Weber P., Bendich A., Schalch W. (1996). Vitamin C and human health—A review of recent data relevant to human requirements. Int. J. Vitam. Nutr. Res..

[70] Petrie R.J., Doyle A.D., Yamada K.M. (2009). Random versus directionally persistent cell migration. Nat. Rev. Mol. Cell Biol..

[71] Amulic B., Cazalet C., Hayes G.L., Metzler K.D., Zychlinsky A. (2012). Neutrophil function: From mechanisms to disease. Annu. Rev. Immunol..

[72] Foxman E.F., Campbell J.J., Butcher E.C. (1997). Multistep navigation and the combinatorial control of leukocyte chemotaxis. J. Cell Biol..

[73] Johnston C.S., Huang S. (1991). Effect of ascorbic acid nutriture on blood histamine and neutrophil chemotaxis in guinea pigs. J. Nutr..

[74] Lykkesfeldt J., Poulsen H.E. (2010). Is vitamin c supplementation beneficial? Lessons learned from randomised controlled trials. Br. J. Nutr..

[75] Carr A.C., Bozonet S.M., Pullar J.M., Simcock J.W., Vissers M.C. (2013). Human skeletal muscle ascorbate is highly responsive to changes in vitamin c intake and plasma concentrations. Am. J. Clin. Nutr..

[76] Carr A.C., Pullar J.M., Moran S., Vissers M.C.M. (2012). Bioavailability of vitamin C from kiwifruit in non-smoking males: Determination of ‘healthy’ and ‘optimal’ intakes. J. Nutr. Sci..

[77] Savini I., Rossi A., Pierro C., Avigliano L., Catani M.V. (2008). Svct1 and Svct2: Key proteins for vitamin C uptake. Amino Acid.

[78] Scientific Committee on Food, Scientific Panel on Dietetic Products, Nutrition and Allergies (2006). Tolerable Upper Intake Levels for Vitamins and Minerals.

[79] Levine M., Wang Y., Padayatty S.J., Morrow J. (2001). A new recommended dietary allowance of vitamin C for healthy young women. Proc. Natl. Acad. Sci. USA.

[80] Mehmood Z.-T.-N.H., Papandreou D. (2016). An updated mini review of vitamin D and obesity: Adipogenesis and inflammation state. Open Access Maced. J. Med. Sci..

[81] Khatami M. (2009). Inflammation, aging, and cancer: Tumoricidal versus tumorigenesis of immunity. Cell Biochem. Biophys..

[82] Hemila H., Chalker E. (2013). Vitamin C for preventing and treating the common cold. Cochrane Database Syst. Rev..

[83] Moser U. (2012). Vitamins—Wrong approaches. Int. J. Vitam. Nutr. Res..

[84] Thompson M., Vodicka T.A., Blair P.S., Buckley D.I., Heneghan C., Hay A.D., Team T.P. (2013). Duration of symptoms of respiratory tract infections in children: Systematic review. BMJ.

[85] Frei B. (2012). Authors perspective—What is the optimum intake of Vitamin C. Crit. Rev. Food. Sci. Nutr..

[86] Moser M.A., Chun O.K. (2016). Vitamin C and heart health: A review based on findings from epidemiologic studies. Int. J. Mol. Sci..

[87] Ross R. (1999). Atherosclerosis—An inflammatory disease. N. Engl. J. Med..

[88] Nyyssonen K., Parviainen M.T., Salonen R., Tuomilehto J., Salonen J.T. (1997). Vitamin C deficiency and risk of myocardial infarction: Prospective population study of men from eastern Finland. BMJ.

[89] Khaw K.T., Bingham S., Welch A., Luben R., Wareham N., Oakes S., Day N. (2001). Relation between plasma ascorbic acid and mortality in men and women in epic-norfolk prospective study: A prospective population study. European prospective investigation into cancer and nutrition. Lancet.

[90] Singh R.B., Ghosh S., Niaz M.A., Singh R., Beegum R., Chibo H., Shoumin Z., Postiglione A. (1995). Dietary intake, plasma levels of antioxidant vitamins, and oxidative stress in relation to coronary artery disease in elderly subjects. Am. J. Cardiol..

[91] Eichholzer M., Stahelin H.B., Gey K.F. (1992). Inverse correlation between essential antioxidants in plasma and subsequent risk to develop cancer, ischemic heart disease and stroke respectively: 12-Year follow-up of the prospective Basel study. EXS.

[92] Sahyoun N.R., Jacques P.F., Russell R.M. (1996). Carotenoids, vitamins C and E, and mortality in an elderly population. Am. J. Epidemiol..

[93] Langlois M., Duprez D., Delanghe J., De Buyzere M., Clement D.L. (2001). Serum vitamin C concentration is low in peripheral arterial disease and is associated with inflammation and severity of atherosclerosis. Circulation.

[94] Lachance P., Langseth L. (1994). The rda concept: Time for a change?. Nutr. Rev..

[95] Harrison F.E. (2012). A critical review of vitamin C for the prevention of age-related cognitive decline and Alzheimer’s disease. J. Alzheimer's Dis..

[96] De Bruijn R.F.A.G., Ikram M.A. (2014). Cardiovascular risk factors and future risk of alzheimer’s disease. BMC Med..

[97] Tramutola A., Lanzillotta C., Perluigi M., Butterfield D.A. (2016). Oxidative stress, protein modification and alzheimer disease. Brain Res. Bull..

[98] World Health Organization (2003). Diet, Nutrition and the Prevention of Chronic Diseases.

[99] Birlouez-Aragon I., Saavedra G., Tessier F.J., Galinier A., Ait-Ameur L., Lacoste F., Niamba C.-N., Alt N., Somoza V., Lecerf J.-M (2010). A diet based on high-heat-treated foods promotes risk factors for diabetes mellitus and cardiovascular diseases. Am. J. Clin. Nutr..

[100] Max Rubner-Institut Nationale Verzehrsstudie II. Ergebnisbericht, Teil 2. http://www.was-esse-ich.de/uploads/media/NVSII_Abschlussbericht_Teil_2.pdf.

[101] Manios Y., Moschonis G., Grammatikaki E., Mavrogianni C., van den Heuvel E.G.H.M., Bos R., Singh-Povel C. (2015). Food group and micronutrient intake adequacy among children, adults and elderly women in greece. Nutrients.

[102] Schupbach R., Wegmuller R., Berguerand C., Bui M., Herter-Aeberli I. (2017). Micronutrient status and intake in omnivores, vegetarians and vegans in Switzerland. Eur. J. Nutr..

[103] Clarys P., Deliens T., Huybrechts I., Deriemaeker P., Vanaelst B., De Keyzer W., Hebbelinck M., Mullie P. (2014). Comparison of nutritional quality of the vegan, vegetarian, semi-vegetarian, pesco-vegetarian and omnivorous diet. Nutrients.

[104] Krebs-Smith S.M., Guenther P.M., Subar A.F., Kirkpatrick S.I., Dodd K.W. (2010). Americans do not meet federal dietary recommendations. J. Nutr..

[105] Murphy M.M., Barraj L.M., Spungen J.H., Herman D.R., Randolph R.K. (2014). Global assessment of select phytonutrient intakes by level of fruit and vegetable consumption. Br. J. Nutr..

[106] Bundesamt für Lebensmittelsicherheit und Veterinärwesen Zu viel Gewicht, zu Wenig Früchte und Gemüse. https://www.blv.admin.ch/blv/de/home/dokumentation/nsb-news-list.msg-id-64373.html.

[107] Hall J.N., Moore S., Harper S.B., Lynch J.W. (2009). Global variability in fruit and vegetable consumption. Am. J. Prev. Med..

[108] Dubuisson C., Lioret S., Touvier M., Dufour A., Calamassi-Tran G., Volatier J.-L., Lafay L. (2010). Trends in food and nutritional intakes of french adults from 1999 to 2007: Results from the inca surveys. Br. J. Nutr..

[109] US Department of Agriculture, Agricultural Research Service, Nutrient Data Laboratory (2015). USDA National Nutrient Database for Standard Reference, Release 28.

[110] Elmadfa I., Meyer A., Nowak V., Hasenegger V., Putz P., Verstraeten R., Remaut-DeWinter A.M., Kolsteren P., Dostalova J., Dlouhy P. (2009). European Nutrition and Health Report 2009.

[111] Deutsche Gesellschaft für Ernährung e. V. (2012). 12. Ernährungsbericht 2012.

[112] Deutsche Gesellschaft für Ernährung e. V. (2008). Ernährungsbericht 2008.

[113] Roman Vinas B., Ribas Barba L., Ngo J., Gurinovic M., Novakovic R., Cavelaars A., de Groot L.C., Van‘t Veer P., Matthys C., Serra Majem L. (2011). Projected prevalence of inadequate nutrient intakes in Europe. Ann. Nutr. Metab..

[114] Troesch B., Hoeft B., McBurney M., Eggersdorfer M., Weber P. (2012). Dietary surveys indicate vitamin intakes below recommendations are common in representative western countries. Br. J. Nutr..

[115] Ministry of Health Labour and Welfare (2008). The Japan National Health and Nutrition Survey 2008.

[116] Kim J., Choi Y.-H. (2016). Physical activity, dietary vitamin C, and metabolic syndrome in the Korean adults: The Korea national health and nutrition examination survey 2008 to 2012. Public Health.

[117] Bailey R.L., Fulgoni V.L., Keast D.R., Dwyer J.T. (2011). Dietary supplement use is associated with higher intakes of minerals from food sources. Am. J. Clin. Nutr..

[118] Fulgoni V.L., Keast D.R., Bailey R.L., Dwyer J. (2011). Foods, fortificants, and supplements: Where do Americans get their nutrients?. J. Nutr..

[119] Skeie G., Braaten T., Hjartåker A., Lentjes M., Amiano P., Jakszyn P., Pala V., Palanca A., Niekerk E.M., Verhagen H. (2009). Use of dietary supplements in the European prospective investigation into cancer and nutrition calibration study. Eur. J. Clin. Nutr..

[120] Nagy S. (1980). Vitamin C contents of citrus fruit and their products: A review. J. Agric. Food Chem..

[121] Vanderslice J.T., Higgs D.J. (1991). Vitamin C content of foods: Sample variability. Am. J. Clin. Nutr..

[122] Marti N., Mena P., Canovas J.A., Micol V., Saura D. (2009). Vitamin C and the role of citrus juices as functional food. Nat. Prod. Commun..

[123] Cahill L., Corey P.N., El-Sohemy A. (2009). Vitamin C deficiency in a population of young Canadian adults. Am. J. Epidemiol..

[124] Hampl J.S., Taylor C.A., Johnston C.S. (2004). Vitamin C deficiency and depletion in the United States: The third national health and nutrition examination survey, 1988 to 1994. Am. J. Public Health.

[125] Coleman-Jensen A., Nord M., Singh A. (2013). Household Food Security in the United States in 2012.

[126] World Health Organization Fact Sheet No. 311: Obesity and Overweight. http://www.who.int/mediacentre/factsheets/fs311/en/.

[127] EFSA NDA Panel (2009). Scientific opinion on the substantiation of health claims related to vitamin C and protection of DNA, proteins and lipids from oxidative damage (ID 129, 138, 143, 148), antioxidant function of lutein (ID 146), maintenance of vision (ID 141, 142), collagen formation (ID 130, 131, 136, 137, 149), function of the nervous system (ID 133), function of the immune system (ID 134), function of the immune system during and after extreme physical exercise (ID 144), non-haem iron absorption (ID 132, 147), energy-yielding metabolism (ID 135), and relief in case of irritation in the upper respiratory tract (ID 1714, 1715) pursuant to article 13(1) of regulation (EC) No. 1924/2006. EFSA J..

